# Correction: Changes in health-related lifestyles and food insecurity and its association with quality of life during the COVID-19 lockdown in Malaysia

**DOI:** 10.1186/s12889-022-13640-9

**Published:** 2022-06-29

**Authors:** Aryati Ahmad, Mohd Razif Shahril, Nadiah Wan-Arfah, Wan Azdie Mohd Abu Bakar, Carmen Piernas, Pei Lin Lua

**Affiliations:** 1 Faculty of Health Sciences, Universiti Sultan Zainal Abidin, Kampus Gong Badak, 21300 Kuala Nerus, Terengganu Malaysia; 2 Nufeld Department of Primary Care Health Sciences, Radclife Primary Care Building, University of Oxford, Oxford, OX2 6GG UK; 3 Centre for Healthy Ageing & Wellness (HCARE), Faculty of Health Sciences, Universiti Kebangsaan Malaysia, Jalan Raja Muda Abdul Aziz, 50300 Kuala Lumpur, Malaysia; 4 Department of Nutrition Sciences, Kulliyyah of Allied Health Sciences, International Islamic University Malaysia, 25200 Kuantan, Pahang Malaysia; 5 Faculty of Pharmacy, Universiti Sultan Zainal Abidin, Kampus Besut, 22200 Besut, Terengganu Malaysia

**Correction: BMC Public Health 22, 1150 (2022)**

**https://doi.org/10.1186/s12889-022-13568-0**

The original publication of this article [1] contained a duplicated author: **Wan Azdie Mohd Abu Bakar** & **Abu Bakar.** The article has been updated to remove this duplication.
